# Changes in Medical Management of Inflammatory Bowel Disease and Reducing Surgical Risk: Investigating Causality Through the Bradford-Hill Criteria

**DOI:** 10.3390/jcm14113824

**Published:** 2025-05-29

**Authors:** Daniela Maggi, Claudio Papi, Stefano Festa, Annalisa Aratari

**Affiliations:** Inflammatory Bowel Disease Unit, San Filippo Neri Hospital, 00135 Rome, Italy; daniela.maggi@aslroma1.it (D.M.); cpapi09@gmail.com (C.P.); stefano.festa@aslroma1.it (S.F.)

**Keywords:** inflammatory bowel disease, surgery, therapy, anti-TNFα, management

## Abstract

**Background**: Inflammatory bowel diseases (IBDs) are chronic progressive conditions, and their management has evolved over time, not only in the number of available medications but also in therapeutic strategies, resulting in a paradigm shift from treat-on-flare to treat-to-target, with the ultimate goal of modifying disease course. Several studies have shown a reduction in the risk of surgery associated with the concomitant increase in anti-tumor necrosis factor α (TNFα) drug prescription, thus inferring a positive impact of anti-TNFα therapy on IBD natural history. However, establishing a causal relationship is complex, as multiple factors influence disease progression. **Methods**: To investigate this relationship, a narrative review applying the Bradford-Hill criteria to the existing literature has been conducted. **Results**: The potential causal link between the introduction and increased use of biologic drugs, particularly anti-TNFα agents, and the reduction in surgical risk in patients affected by IBD are critically reviewed. **Conclusions**: Establishing a direct causal link between increased anti-TNFα prescriptions and long-term outcomes remains a difficult issue. Multiple factors like greater awareness, early diagnosis, multidisciplinary approaches, introduction of guidelines, and ongoing education also contribute to improved prognosis.

## 1. Introduction

Inflammatory bowel diseases (IBDs) are chronic conditions characterized by a relapsing–remitting course. Despite their fluctuating nature, these diseases often exhibit a progressive disabling course and adversely affecting patients’ quality of life. Managing IBD presents considerable challenges, necessitating the involvement of dedicated healthcare professionals and a multidisciplinary approach to address potential complications and extraintestinal manifestations. Over the past three decades, treatment options for IBD have significantly evolved. Initially, the introduction of anti-tumor necrosis factor α (TNFα) agents marked a pivotal advancement, followed by the development of second- and third-generation biologics, the emergence of biosimilars, and more recently, the introduction of small molecules. Concurrently, the expansion of available therapies has prompted a shift in treatment strategies from the traditional “treat-on-flare” model to a “treat-to-target” approach, as recommended by the Selecting Therapeutic Targets in Inflammatory Bowel Disease (STRIDE) and Selecting End PoInts foR Disease-ModIfication Trials (SPIRIT) recommendations [1,2,3]. The ultimate goal of these strategies is to modify the disease course, prevent long-term complications and the need for surgery, reduce disability, and ultimately enhance patients’ quality of life [4].

The concept of remission, as defined by the STRIDE and SPIRIT consensus, encompasses clinical criteria (resolution of abdominal pain, rectal bleeding, and diarrhea), endoscopic criteria (absence of ulcerations in Crohn’s disease and a Mayo score of 0–1 in ulcerative colitis), and patient-reported outcomes (resolution of abdominal pain, normalization of bowel movements, and improvement in quality of life). Achieving remission at various levels has been correlated with enhancements in several long-term outcomes, including hospitalizations, surgeries, disability, cancer incidence, and mortality [5,6,7]. Among these outcomes, surgical risk is the most straightforward to study and is frequently evaluated for several reasons: it is well defined, relatively common, and does not necessitate an excessively long follow-up period compared to other significant but less frequent endpoints (such as mortality or cancer) or endpoints that lack universally accepted definitions, like disability. Studying the relationship between therapies and surgical risk in IBD is essential. As surgery remains a key component in managing IBD complications, understanding how therapies influence surgical risk is critical for optimizing timing, minimizing complications, and improving patient outcomes.

Numerous studies have demonstrated a decline over time in the risk of surgery, which has been associated with an increase in the prescription of anti-TNFα drugs, suggesting a positive impact of anti-TNFα therapy on the natural history of IBD. For instance, a retrospective study by Rungoe et al. analyzed the clinical histories of 48,967 IBD patients from 1979 to 2011, examining the type of therapy and the associated risk of surgery using Cox regression analysis [5]. They found that the cumulative risk of surgery at 5 years decreased from 44.7% in the 1979–1986 cohort to 19.6% in the 2003–2011 cohort for Crohn’s disease (CD), and from 11.7% to 7.5% for ulcerative colitis (UC). Concurrently, there was an increase in the use of thiopurines and anti-TNFα agents compared to aminosalycilates and corticosteroids. Similar findings have been reported in other studies, including those involving pediatric populations, for both UC and CD [6,7,8,9,10,11,12,13].

When a statistical association is identified in clinical research, the subsequent step is to determine whether this association is causal or not. Indeed, there are various types of statistical associations: an association may be spurious or false due to systematic errors (e.g., selection bias, information bias) or random errors; there may also be an indirect association that is real but not causal, influenced by confounding factors that were not accounted for in the analysis. When speculating on the potential link between a reduction in surgical risk and an increased prescription of anti-TNFα, it is unlikely that a spurious association exists, as the findings are derived from well-conducted population studies characterized by large sample sizes, clearly defined diagnostic criteria, explicit endpoints, and extended follow-up periods, making systematic bias improbable. Random error is also unlikely, given that multiple studies from different countries arrive at the same conclusion. However, it is conceivable that an indirect association exists due to confounding factors. For instance, coffee consumption may appear to be associated with the development of lung cancer, when in fact it is correlated with cigarette smoking, which is the actual risk factor for lung cancer [14].

Several strategies can be employed to control for confounding factors: randomization, restriction, or matching for confounding variables, or, following data collection, the use of multivariate models or propensity score-based analyses. However, these corrections only address known confounding factors, while unknown confounding variables cannot be accounted for. For example, the study by Ramadas et al. retrospectively analyzed the cases of 341 patients with CD, revealing a progressive increase in the use of immunosuppressants: 11% in the 1986–1991 cohort, 28% in the 1992–1997 group, and 45% in the 1998–2003 group (*p* < 0.001) [11]. Additionally, there was a significant reduction in long-term steroid use (44%, 31%, and 19%, respectively, *p* < 0.001) and a decrease in the cumulative probability of surgery (59%, 37%, and 25%, respectively, *p* < 0.001). However, while the study assessed the percentage of smokers at diagnosis, it did not track how many of these individuals continued to smoke during follow-up. Given the established role of smoking in IBD outcomes, a reduction in the percentage of smokers could potentially act as an unknown confounding factor affecting the results. The study by Nguyen et al. illustrates how considering additional factors complicates the determination of causality in this association [12]. In their retrospective design, they analyzed 3403 patients with CD diagnosed between 1988 and 2008 in Canada, dividing them into three cohorts: cohort 1 (before 1996), cohort 2 (1996–2000), and cohort 3 (2001 onwards). They compared the risk of surgery, hospitalization, use of immunomodulators, and access to specialist care. The results indicated a progressive reduction in the risk of surgery across the three cohorts (30%, 22%, and 18%, respectively). Additionally, there was an increased prevalence of specialist visits one year after diagnosis in cohorts II and III compared to cohort I (53%, 72%, and 88%, respectively; *p* < 0.0001), and this increase was associated with a reduced need for surgery (HR 0.83; 95% CI, 0.71–0.98). The use of immunomodulators in the first year following diagnosis also increased over time (11% in cohort II vs. 20% in cohort III; *p* < 0.0001). Several factors, in fact, may contribute to surgical risk, as described more recently [15].

One way to eliminate confounding factors in retrospective studies is to use multivariate analyses and propensity score matching. An example of this is the study by Jeuring et al. [6], which retrospectively analyzed 1162 patients with CD, dividing them into three cohorts: 1991–1998, 1999–2005, and 2006–2011. The study examined drug exposure, hospitalization rates, surgery rates, and progression phenotypes using Kaplan–Meier curves, followed by comparisons with Cox regression. Ultimately, propensity score matching was employed to establish the relationship between pharmacological exposure and long-term outcomes. This study found an increase in exposure to immunomodulators over time (from 30.6% in 1991–1998 to 70.8% in 2006–2011), alongside a significant reduction in hospitalization rates (from 65.9% to 44.2%) and surgery rates (from 42.9% to 17.4%). However, these improvements were not statistically associated with each other in multivariate analyses, suggesting the presence of other factors influencing the changes in disease progression.

We can conclude that establishing causal associations requires careful consideration. To aid in this, Sir Bradford-Hill proposed nine criteria in 1960 to assess the credibility of causal associations (Table 1) [16,17,18,19,20,21,22]. 

The first key criterion is temporal sequence: exposure must occur before the outcome. This is hard to establish in chronic diseases due to multiple factors over time. Second, the strength of association (e.g., relative risk, odds ratio) supports causality if strong, while weak associations may reflect bias. Third, consistency across studies and populations strengthens causal inference. Fourth, a biological gradient or dose–response relationship, like smoking and lung cancer, supports causality. Fifth, specificity suggests that a single exposure leads to one outcome, though this is rare and a weaker criterion. Sixth, a biological mechanism linking exposure and outcome adds plausibility, though is limited by current knowledge. Seventh, coherence with existing knowledge supports causality. Eighth, experimental evidence (e.g., clinical trials) provides strong support. Ninth, analogy with known causal relationships may help but can mislead. As we have seen, while these criteria are widely used, they are not without criticism. Bradford-Hill himself referred to them not as strict criteria but as nine different perspectives from which we should examine associations before concluding causality [16]. He argued that these perspectives do not provide indisputable evidence for or against a cause–effect hypothesis, but they can help us weigh the evidence for or against a potential causal interpretation.

We performed a systematic literature search to identify studies published between 1998 (the year anti-TNFα therapy was first introduced for IBD) and 2024. Our aim was to retrieve evidence addressing each of the nine Bradford-Hill criteria for causation in relation to the impact of anti-TNFα agents on surgical risk in IBD. Inclusion criteria were original cohort studies reporting surgical outcomes in IBD populations exposed to anti-TNFα therapies, clear specification of study timeframe, and adequate reporting of demographic and clinical characteristics. We also noted whether statistical methods such as multivariable regression, propensity score matching, or time trend analysis were employed to adjust for confounding factors or temporal effects.

The purpose of this narrative review is to explore the possible causal relationship between changes in medical treatment and the reduction in the risk of surgery using the Bradford-Hill criteria—an approach that has not been previously applied to this specific topic.

## 2. Relevant Sections

### 2.1. Criterion No. 1—Temporal Sequence: Does an Increase in Anti-TNFα Prescription Truly Precede the Reduction in Surgical Risk?

A meta-analysis conducted by Tsai et al. in 2022 examined surgical rates and trends in UC and CD, incorporating data from 44 cohort studies spanning from 1962 to 2016 for UC and from 1955 to 2015 for CD [23]. The analysis revealed a decreasing trend in both short- and long-term surgical risk, with a reduction of 25–50% in patients diagnosed with IBD after 2000 compared to those diagnosed in earlier decades. While it appears that exposure precedes the outcome, the authors did not specify the exact factors contributing to this reduction in surgical risk, suggesting a multifactorial influence. Notably, a prior meta-analysis by Frolkis et al. in 2013 [24] indicated that surgical risk had already been on a linear decline since the 1960s. This trend was corroborated by subsequent studies, including those by Qvist et al. in 2021 [25], Murthy et al. in 2019 [26], and Osei in 2021 [27], which demonstrated that the introduction and gradual adoption of anti-TNFα therapies coincided with an existing downward trend, without significantly altering its trajectory. Therefore, while a temporal relationship between anti-TNFα introduction and reduced surgical rates exists, the evidence does not clearly establish a causal or leading role for these therapies in the observed decline. Other concurrent factors are likely contributing, and further investigation is needed to delineate their relative impact [24,25,26,27].

### 2.2. Criterion No. 2—Strength of Association: Is the Relative Risk Reduction or Odds Ratio Statistically Significant?

A strong association is typically defined as a relative risk of greater than 3 in cohort studies or an odds ratio exceeding 4 in case–control studies, as these values provide robust support for a causal relationship [28]. In this context, a meta-analysis by Law et al. in 2023 assessed the impact of early versus late exposure to anti-TNFα therapies on surgical risk across 18 studies (3 focused on UC and 15 on CD) [29]. The findings suggest that early treatment with biologics is associated with a lower surgical risk in CD (odds ratio 0.63; 95% confidence interval, 0.48–0.84), although this association lacks high statistical strength. Conversely, the same study indicated an increased odds ratio for UC (OR 2.86, 95% confidence interval, 1.3–6.30), likely attributable to selection bias, as patients with more severe disease were initiated on biologics earlier.

### 2.3. Criterion No. 3—Consistency of Association: Has the Association Been Found in Multiple Studies?

Certainly, there are studies that support this, but not all findings are consistent. For instance, the research conducted by Burisch et al. in 2017 focused on the Epi-IBD cohort, which included unselected patients with CD from 29 European centers, representing a population of over 10 million people [30]. This study followed patients prospectively to analyze disease progression and outcomes five years post-diagnosis. It highlighted a notable difference in the use of immunomodulators between Western and Eastern European countries, with biologics being utilized significantly more in Western Europe (33% vs. 14% in Eastern Europe) and immunomodulators also being more prevalent (66% in Western vs. 54% in Eastern Europe). Despite the earlier and more widespread use of these treatments in the West, the study found no statistically significant differences in five-year surgical outcomes or disease progression phenotypes between patients from the two regions. Similarly, Zhao et al. examined a Danish cohort of over 6000 patients treated with biologics from 2011 to 2018, with 51% diagnosed with UC and 49% with CD [31]. Their findings indicated a gradual increase in the prescription of anti-TNFα therapies (from 5% to 10.8% for UC and from 8.9% to 14.5% for CD) and a decrease in surgery rates for both conditions; however, this reduction was not statistically linked to the use of anti-TNFα therapies.

### 2.4. Criterion No. 4—Biological Gradient: Is There a Dose–Response Relationship?

A clear dose–response relationship has not been established, as standard dosages are typically used. However, some studies suggest that longer treatment durations may correlate with a reduced risk of surgery. For example, Peyrin-Biroulet et al. conducted a retrospective observational study on CD patients diagnosed between 2000 and 2008 at the University Hospital of Nancy, with a median follow-up of 57 months [32]. Their multivariate analysis identified treatment duration with anti-TNFα therapy of less than 16 months (HR = 3.86; 95% CI 1.77 to 8.45) or with azathioprine for less than 1.5 months (HR = 2.00; 95% CI 1.20 to 3.34) as positive predictive factors for surgery. However, this duration–response relationship has not been consistently confirmed. In another study by Eberardson et al. involving 1856 Swedish patients treated with TNFα inhibitors from 2006 to 2014, no statistically significant difference in intestinal resection rates was observed between those who discontinued anti-TNFα therapy for less than 12 months and those who continued for more than 12 months (*p* = 0.27) [33].

### 2.5. Criterion No. 5—Specificity of Association: Does Exposure Lead to a Single Outcome?

This criterion can often be misleading. Everyday experiences show us that a single event can lead to various outcomes, and the presence of one outcome does not rule out the possibility of others. Therefore, this criterion does not significantly strengthen the argument for causality, especially in chronic, complex, and multifactorial inflammatory diseases [19].

For example, cigarette smoking is generally associated with a reduced risk of developing UC. A meta-analysis conducted in 2016 reviewed data from 16 observational studies involving 2615 patients and analyzed outcomes such as disease flare-ups, colectomy rates, disease extension, and pouchitis development [34]. The results indicated that the risk of colectomy (OR = 0.89; 95% CI 0.62–1.26), disease flare (OR = 1.26; 95% CI 0.65–2.44), proximal extension (OR = 0.57; 95% CI 0.20–1.66), and pouchitis development (OR = 0.57; 95% CI 0.21–1.53) was not significantly lower in smokers compared to non-smokers. Therefore, smoking does not appear to enhance the natural history of UC.

### 2.6. Criteria No. 6 and 7—Biological Plausibility and Consistency with Current Knowledge—Does the Association Make Sense in Light of Our Knowledge?

It absolutely does, as it is mediated by the concept of mucosal healing, which is recognized to have a positive impact on various outcomes [35]. Mucosal healing reduces the risk of recurrence, hospitalization, and surgical interventions, as well as the formation of fistulas and post-operative recurrence in CD, and lowers the risk of colorectal cancer in UC. It is important to note that anti-TNFα therapies induced mucosal healing [36].

### 2.7. Criterion No. 8—Is There Experimental Evidence to Support?

Pivotal trials have demonstrated that biologics are effective in inducing and maintaining remission [37,38,39,40]; however, their primary endpoints focus on clinical remission, with most studies having a follow-up period limited to 54 weeks. Surgery is considered a secondary and time-dependent outcome. There are few randomized controlled trials specifically analyzing long-term outcomes, such as surgery or hospitalizations. A meta-analysis by Mao et al. in 2016 included seven RCTs (two for UC and five for CD) published between 1980 and 2016, which examined the effectiveness of biologics or immunomodulators compared to placebo, directly assessing hospitalization and surgery rates [41]. The analysis revealed that in both UC and CD, anti-TNFα therapies significantly reduced hospitalizations (OR 0.46, 95% CI 0.36–0.60) and surgeries (OR 0.23, 95% CI 0.13–0.42) compared to placebo, while a similar reduction was not observed with azathioprine or vedolizumab. However, important limitations must be considered, including the limited number of RCTs, heterogeneity in inclusion criteria, and restricted follow-up, which weaken the robustness of this evidence as well as evidence described more recently [42].

### 2.8. Criterion No. 9—Analogy Criterion: Is the Association Similar to Others?

The analogy criterion provides weak support for causal inference. While the effectiveness of a model in one context may suggest its applicability in another, neither the presence nor the absence of analogy can confirm or exclude causality. For instance, multiple levels of evidence highlight both the association between IBD and other immune-mediated inflammatory diseases (IMIDs—such as rheumatologic, dermatologic, and ocular conditions) and the efficacy of the same advanced therapies (biological agents or small molecules) across different IMIDs [43]. Nevertheless, the success of various biological therapies (in particular, of anti-TNF alpha) in changing the natural history of rheumatological diseases does not necessarily imply their effectiveness in changing the natural history of IBD, where evidence showing a clear reduction in surgery for complications is lacking [44].

## 3. Discussion

The application of the Bradford-Hill criteria does not establish an unequivocal causal relationship between anti-TNFα therapies therapy and improvements in long-term outcomes. This ambiguity likely arises from a multitude of factors beyond the mere administration of biologics. Conversely, it is plausible that various confounding variables may obscure the beneficial role of anti-TNFα therapies in enhancing long-term outcomes, potentially leading to suboptimal performance. For instance, Schoepfer’s survey indicates that many Swiss gastroenterologists primarily base their therapeutic decisions on clinical disease activity rather than on endoscopic findings or biomarkers [45]. This is noteworthy given that mucosal healing is a key endpoint in clinical trials. Evidence suggests that active and early monitoring utilizing endoscopic parameters significantly enhances long-term outcomes in both CD and UC [46]. Moreover, the prescription patterns for anti-TNFα therapies appear to remain limited. Siegel et al. conducted a retrospective analysis involving 28,119 UC patients and 16,260 CD patients, revealing that 5-ASA therapy continues to dominate treatment regimens: 61% of UC patients and 35% of CD patients were initiated on 5-ASA monotherapy as first-line treatment [47]. This was followed by steroid monotherapy (25% for UC versus 42% for CD), while less than 1% of UC patients and fewer than 5% of CD patients received biologics as first-line therapy. Among those not initially treated with anti-TNFα therapies, only 6% of UC patients and 19% of CD patients transitioned to biologic treatments later on. These prescribing trends persist despite established guidelines recommending anti-TNFα therapies as top-down approaches—either alone or in combination—for all patients with moderate to severe disease. Furthermore, the use of 5-ASA in the management of CD has been removed from the recommendations, emphasizing that steroids should serve merely as a bridge to other immunomodulatory or biological therapies. The enhancement in prognosis cannot be attributed solely to the rise in prescriptions of this drug class; rather, it results from a combination of multiple factors. These include heightened interest and awareness regarding IBD, earlier diagnosis and management by gastroenterologists, multidisciplinary approaches, the introduction of practical guidelines, and the promotion of continuous medical education. Despite significant advancements in treatment and management, our most effective medications and evidence-based strategies are utilized in only a small percentage of patients. This indicates the existence of barriers that hinder optimal patient management. The findings of this narrative review have significant implications for clinical practice, particularly in how treatment strategies for IBD patients are developed and refined. While it is well established that advances in medical management, particularly the use of biologic therapies, have contributed to reducing surgical risks, these findings suggest that the timing and approach to biologic therapy should be carefully considered in the context of each patient’s individual disease course.

In interpreting these findings, it is essential to consider key limitations of this narrative review. Notably, in the available literature, there is a lack of randomized controlled trials (RCTs) specifically examining the impact of anti-TNFα therapies on surgical outcomes in IBD. Most of the existing data are derived from observational studies, which are inherently subject to confounding and cannot establish causality. Additionally, selection bias may influence treatment outcomes, as patients prescribed biologics often differ in baseline characteristics such as disease severity, timing of intervention, and access to specialized care. These methodological constraints must be acknowledged when evaluating the true effectiveness of anti-TNFα therapies in altering long-term clinical trajectories, including the need for surgery. A more in-depth analysis of potential confounding variables that may affect the relationship between anti-TNFα therapy and surgical risk would be valuable. For example, the influence of concomitant non-biologic therapies may modify outcomes and obscure the isolated effect of anti-TNFα therapy. Temporal changes in diagnostic and therapeutic practices, including earlier disease detection, increased use of endoscopic monitoring, and shifts in treatment thresholds, could independently affect surgical rates over time; for example, more sensitive endoscopic assessments have allowed for earlier intervention and more precise monitoring of disease activity, which may influence surgical decisions. A limitation of this work is the lack of data on economic factors influencing the IBD management and surgical outcomes across regions. Economic disparities can lead to variations in access to biologic therapies, with limited-resource settings facing delays or restrictions that may increase surgical risk. In contrast, better-resourced healthcare systems allow for earlier access to advanced treatments, potentially reducing surgery rates [48]. Future studies should consider economic disparities, as they influence treatment access and clinical decisions. Addressing these differences is key to developing equitable strategies and improving global outcomes.

Further research, therefore, is still needed to clarify the role of biologic therapies in modifying surgical risk in IBD. Large-scale and long-term prospective trials are essential and should specifically focus on the timing of biologic initiation and the duration of therapy before surgery, as well as explore the role of concomitant therapies and their potential interaction with biologic treatments. Studies should also investigate how advances in diagnosis and early intervention influence the timing of surgery and clinical decision-making. Understanding the combined impact of the many variables will be critical to optimizing treatment strategies for IBD patients.

## 4. Conclusions

In summary, the current evidence suggests that anti-TNFα therapies may play a role in reducing surgical risk in IBD; however, this relationship is influenced by multiple clinical and systemic factors. While biologics have changed the treatment landscape, their real-world impact remains shaped by timing of initiation, treatment strategies, and healthcare delivery practices. The findings of this review support a more individualized, early-intervention approach, and highlight the need to close the gap between guideline recommendations and clinical practice.

## Figures and Tables

**Table 1:** Bradford-Hill Criteria [16].

(1) Temporality (2) Strength (3) Biological gradient (4) Consistency (5) Specificity (6) Plausibility (7) Coherence (8) Analogy (9) Experiment
